# Processing laser ablated plasmonic nanoparticle aerosols with nonthermal dielectric barrier discharge jets of argon and helium and plasma induced effects

**DOI:** 10.1038/s41598-022-27294-5

**Published:** 2023-01-03

**Authors:** Taj Muhammad Khan, Gustavo Andrade Silva Alves, Amjad Iqbal

**Affiliations:** 1 School of Physics and CRANN, Trinity College Dublin, The University of Dublin, Dublin 2, Ireland; 2 National Institute of Lasers and Optronics College, Pakistan Institute of Engineering and Applied Sciences, Nilore, Islamabad 45650 Pakistan; 3 Department of Materials Technologies, Faculty of Materials Engineering, Silesian University of Technology, 44-100 Gliwice, Poland; 4 Department of Mechanical Engineering, CEMMPRE- Centre for Mechanical Engineering, Materials and Processes, University of Coimbra, Rua Luis Reis Santos, 3030-788 Coimbra, Portugal

**Keywords:** Materials science, Nanoscience and technology, Physics

## Abstract

Dielectric-barrier-discharge (DBD) plasma jets provide viable state-of-the-art nonthermal processes for a wide range of nanomaterials including particle transport and deposition. We report the interaction of argon and helium plasma jets with the particle aerosol, produced by ns laser ablation of a silver target and subsequently their transport for deposition on a distant substrate. The nanofeatures and functionality of the nanoparticles, entrained and deposited with the two plasma jets were compared using high-resolution electron microscopy, helium ion microscopy, scanning electron microscopy, ultraviolet–visible spectroscopy, and in terms of the SERS effect. The plasma jet facilitates the transport of the particle aerosol under the upshot of plasma ionic wind, caused by the high electric field in the plasma. Compared to the helium plasma jet, the argon plasma jet leads to a relatively large particle deposition and promotes the formation of aggregates. The helium plasma jet enabled the deposition of spatially well dispersed particles. In both cases, the deposited particle was crystalline and plasmonic active. The plasma-driven altered morphology, expedient particle transport, and formation of agglomerates or spatially well dispersed particles are explained in plasma-induced ionic-wind, and dusty plasma framework. The findings are novel and interesting from the perspective of plasma–surface deposition, surface nanoengineering, and nanomaterial processing for applications in sensing, catalysis, surgical tools, futuristic coating technology, and heat-sensible biological activities.

## Introduction

The recent tremendous progress in plasma assist surface coating, surface nanoengineering, and proven understanding of the physical phenomena in atmospheric pressure dielectric barrier discharge (DBD) jets has generated a growing interest in the plasma-material frontier. The scientific and technological concern in these plasmas arose more than 70 years back, and since then DBDs have been recognised a great asset to humans for innovative and industrially feasible cutting-edge technologies. For the fraternity of scientists and engineers, DBDs are offering several potential solutions to everyday problems with the freedom of working in an open and flexible workplace^1–5^. It is now recognised a platform technology, replacing and/or integrating with the production stages in the growing medicine and healthcare, surface activation and modification, biomedical and surgical, textiles, sterilized food processing and packaging, smart agriculture, clean energy, and environmental remediation, ground-breaking materials, microfabrication, and many more^2–23^. From plasma-created high-purity nanocrystals to plasma-driven ultraviolet lights, it has great promise for active plasmonic nanostructures, active surfaces covered with graphene, beyond graphene 2D-functional materials, and recently in COVID-19 inactivation^2,6,14,23–26^.

Typical DBD jets are generated in inert or reactive gases and/or a combination of both under a high-voltage ac or pulsating-dc at frequencies of Hz to MHz^2–6,23,25^. At least one of the electrodes is shielded with a dielectric material to limit the discharge current and thermal instabilities. For these plasmas, ${\text{T}}_{{{\text{electron}}}} { }\left( {10,{ }000{ }{-}{ }100,{ }000{\text{ K}}} \right) > > {\text{ T}}_{{{\text{ion}}}} ,{\text{ T}}_{{{\text{neutral}}}} { }\left( {300{\text{ K}}} \right){\text{ with T}}_{{\text{ion}}} \approx {\text{T}}_{{\text{neutral}}}$, ${\text{T}}_{{{\text{gas}}}} = 300 - 800{\text{ K}}$, and a typical low-degree of ionisation ${\upchi } < 10^{ - 3} - 10^{ - 8} { }$^2,6^. The outflowing plasma jets from the discharge are electrically driven phenomena; comprising of small plasma bullets (ionisation wavefronts), propagating at speeds of 10^4^–10^7^ ms^−1^^2,23,25^. Such plasma schemes provide efficient ways to generate distantly transferred and adjustable plasma volumes of high charge density and are well described by comprehensive review reports^2,6,27,28^.

The plasma jets excited in atomic argon and helium are more frequently used to achieve energy-intensive and effective gas-phase chemistry without the need for elevated gas temperature, and transporting energetic charge species to the target site^2,6^. Typically, these jets are characterized by electron charge density (n_e_) ≤ 10^13^ cm^−3^ and electron temperature (T_e_) ⁓ 0.5–10 eV^2,6,23,25^. Notably, a helium jet can be launched either way by setting the live electrode (attached to the ac high-voltage terminal) upstream and the ground electrode downstream of the gas feed tube or mutually swapping the two electrodes. While in the case of argon, it only emits if the live electrode sets upstream and the ground electrode downstream of the reactor tube^28,29^.

A wide variety of nanomaterials have been produced with DBD plasma jets using source precursors^2,13–24^. In the plasma interaction, the source precursors effectively dissociate to produce reactive radicals and ions and heat up the nanomaterial by reacting on the particle surface exothermally. Nanoparticles of platinum and gold were prepared with spatially confined atmospheric pressure plasma jets (APPJs) of hydrogen and helium respectively^21,24^. Similarly, APPJs were applied for direct nanoparticle coating of silver and palladium on silicon wafers, fabrics, and Al_2_O_3_ (sapphire) for antibacterial and catalysis applications^22^. Owing to unique plasma-material interaction, future ultrathin materials such as doped graphene, reduced graphene (rGO), and beyond graphene 2D –materials (h-BN, MoS_2_) could be made for super energy harvesting devices like supercapacitors and broad-band photodetectors^2,14^.

An important feature of DBDs is the interaction of the active plasma species with the nanostructures with the effect to prevent agglomerates formation due to particle charging^6^. Such nanomaterials are highly desired for specific applications such as nanosilver for antibiotic in marine toxicology^30^. In surface science, the transferred plasma jets of DBDs provide reasonable solutions for several problems including surface activation (for better bonding), functionalities, modification (for hydrophobicity, hydrophilicity), and surface etching (for patterning)^2,6^. Favourable plasma jet conditions can be established to control the process of plasma-particle interaction to make surfaces, covered by dispersed nanoparticles and/or assembled nanostructures. A surface with assembled nanostructures offers better chemical sensing in surface-enhanced Raman scattering (SERS) by amplifying the SERS signals due to the ‘hot sites’^31,32^. Integrating transferred discharge jets with the physical process of a nanomaterial such as a laser ablation is of great material business in surface science: this is beneficial in resolving the particle loss of chemical precursors in the active discharge region surrounded by the electrodes. For laser ablation at atmospheric gas pressure, a high-density plasma (varying from ne 1.59–2.27 × 10^18^ cm^−3^) is formed under various ambient environments^33,34^. The ablation plume encounters a strong collisional coupling and is captured by the ambient gas to form a nanoparticle aerosol by frequent collisional condensation^35,36^. The particle aerosol so formed can be processed with a plasma jet to deliver it to the substrate for deposition to make active surfaces. This process yields a significant impact on pharmaceutical industry by enhancing the flowability of fine-grained powder to prevent the clogging of apparatus, and precious product losses, and to sustain longer maintenance times^37,38^. The plasma interaction also tunes the particle size, surface morphological nanofeatures, and particle spatial distribution^25,32^. Despite their great potential in material sciences, the scientific literature is lacking such research studies that are of great technological and futuristic importance for direct utilization, particularly in plasma–surface frontier.

In this article, the post-processing of plasmonic nanoparticle aerosols of silver and their subsequent deposition at atmospheric pressure with the gas-ionized media of argon and helium plasma jets is reported. In the procedure, unlike the traditional chemical injection process, high-purity particle aerosols are produced by ns laser ablation process and entrained in the external afterglow (eAG) discharge streams. Favourable condition of the plasma jets that inhibits and/or preferentially facilitates the formation of agglomerates is described in the framework of dusty plasma. The expedient particle deposition in the presence of discharge is explained by the phenomenon of plasma-induced ionic wind. The SERS sensitivity aspect of the agglomerates in the case of argon plasma is compared with the spatially dispersed particles obtained with the helium discharge jet. The underlying knowledge of cold plasma jets from the perspective of processing and deposition of a nanomaterial is useful for nano-coating technology, nano-printing, biomedical, pharmaceutical, catalysis, and sensor applications.

## Experimental procedure

**Figure 1:**
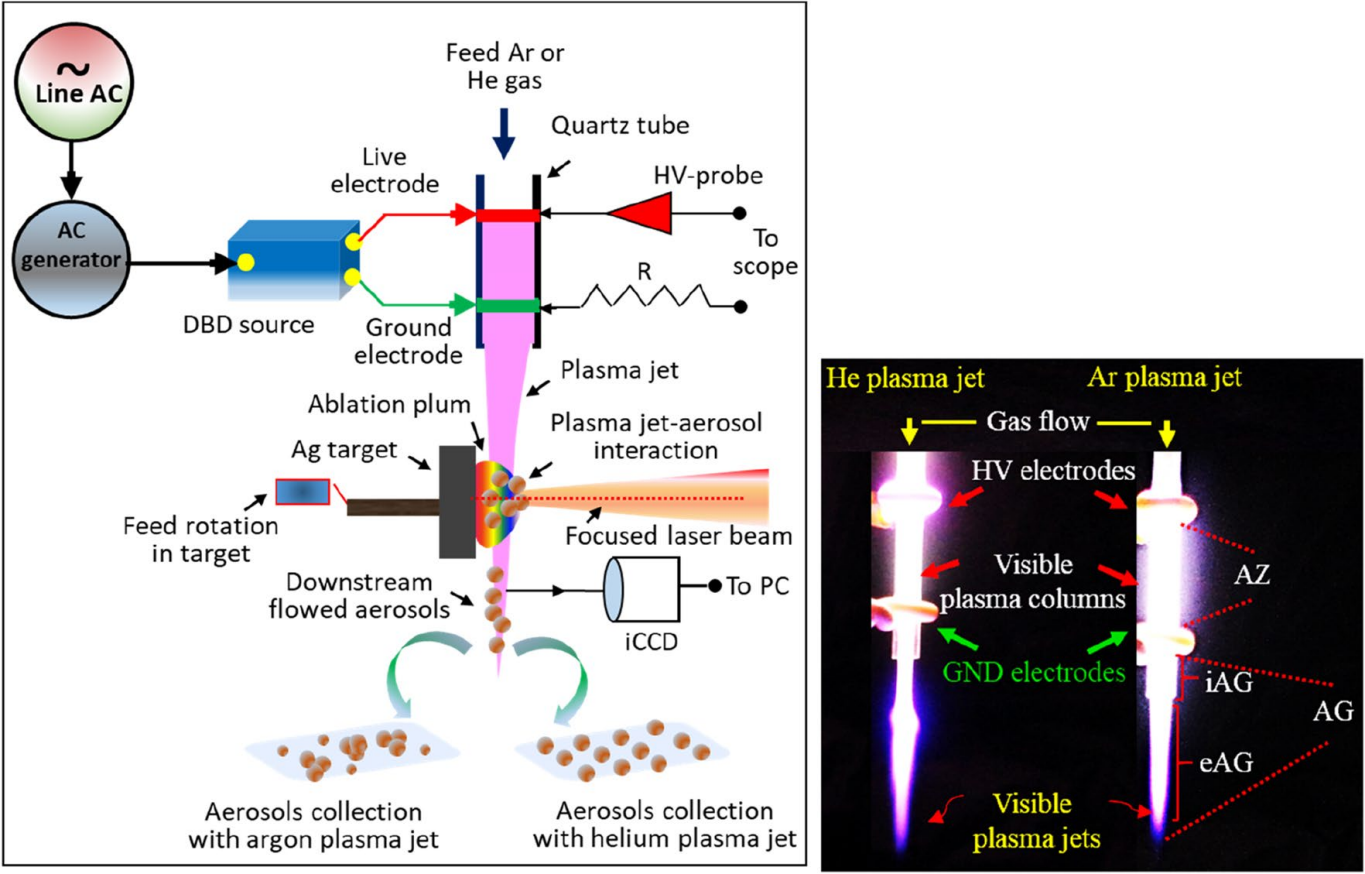
Experimental sketch showing a DBD plasma source, laser ablation process, post-processing & deposition of nanoparticle  aerosols with the outflowing plasma jet, the HV-probe to measure the electrical signals, and iCCD to capture discharge optical images. The insets shown are two digital photographs, illustrating the physical appearance of various regions of argon and helium plasmas; the active zone (AZ) between the electrodes, and the afterglow (AG) region, starts just behind the grounded electrode and is further subdivided as the internal afterglow (iAG) inside the tube (beyond the ground electrode to the tube orifice) and the external afterglow (eAG) outside the tube which propagates as a visible stream.

In the schematic illustration of the experimental setup shown in Fig. 1, plasma jets were excited in the tubular arrangement in atomic argon and helium gases of purity > 99.99%. The photographs in the inset show visible plasma formed in flowing argon (right) and helium (left) upon electrical excitation. Two ring-shaped copper metal electrodes each of thickness of 2 mm were wrapped on the outer surface of the quartz tube (ε = 3.76 @ 1 MHz, 20 °C) of inner and outer diameters of 2.4 and 3 mm respectively, and process gas was flowed at 36 m s^−1^, controlled by a mass flow controller (Key instruments, Model No. FR2A12BVB). The centre-to-centre separation between the electrodes was fixed at 20 mm and the downstream ground electrode was set at 8 mm from the tube nozzle. In the arrangement, direct plasma-electrode contact is avoided to establish a clean plasma environment. The plasma device was operated in the normal mode where the live electrode sets upstream and the ground electrode downstream side of the gas feed tube respectively. Plasma jets were launched outside the tube orifice under electrical excitation by using a high-amplitude sinusoidal voltage of 20 kV at a frequency of 15 kHz (inset in Fig. 1). The jet propagates as visible plume through the ground electrode and get narrowing as penetrates in the ambient air. The plasma jet was set vertically downward to have a direct interaction of the jet with the particle aerosol formed by laser ablation. The process of laser ablation has been described in our previous reports^34,36^. Briefly, a 248 nm, 25 ns excimer laser (Lambda Physik Complex Pro 102 KrF laser system) was used to ablate a silver target, fixed on a dc-driven rotating disk. The laser delivers an average energy of 700 mJ and average power of 7 W at the rep. rate of 10 Hz. With a beam delivery system, the required laser irradiance of 1.4 J cm^−2^ was achieved at the target surface. The area of the laser spot at the focus was 6.28 × 10^−2^ cm^2^. The proposed arrangement allows the plasma jet to entrain silver particle aerosol for deposition onto a distant substrate. The particle aerosol deposited was characterised using FEI Titan transmission electron microscopy (TEM), high-resolution TEM (HRTEM), gas field ion source (GFIS) helium ion microscope (Zeiss ORION plus series), and Carl Zeiss Ultra plus scanning electron microscopy (SEM) to examine the formation of agglomerates, the particle morphology, size, and spatial distribution. Optical absorbance was measured in the spectral range of 300–800 nm using a Cary 50 UV–Vis optical absorption spectrometer. The excitation ac voltage across the electrodes was measured by attaching directly an HV-probe (Tektronix P6015A, 75 MHz, and 1000 X) to the live electrode while the discharge current was obtained by measuring the voltage on the resistor (R = 50 Ω) connected on the ground electrode side (see Fig. 1). The current–voltage waveforms were monitored by using the digital oscilloscope (Tektronix DPO 3034, 200 MHz) at high sensitivity. The optical images of the plasma jets and the plasma active column within the electrodes were acquired with an intensified charge-coupled device (iCCD) (Andor iStar 334 T) using the gate width of 1 µs and exposure time of 3 ms to allow several discharges to capture. The photographs taken with a digital camera with an exposure time of 1/60 s provide the physical appearance of the various visible plasma regions as displayed in the inset of Fig. 1.

## Results

**Figure 2:**
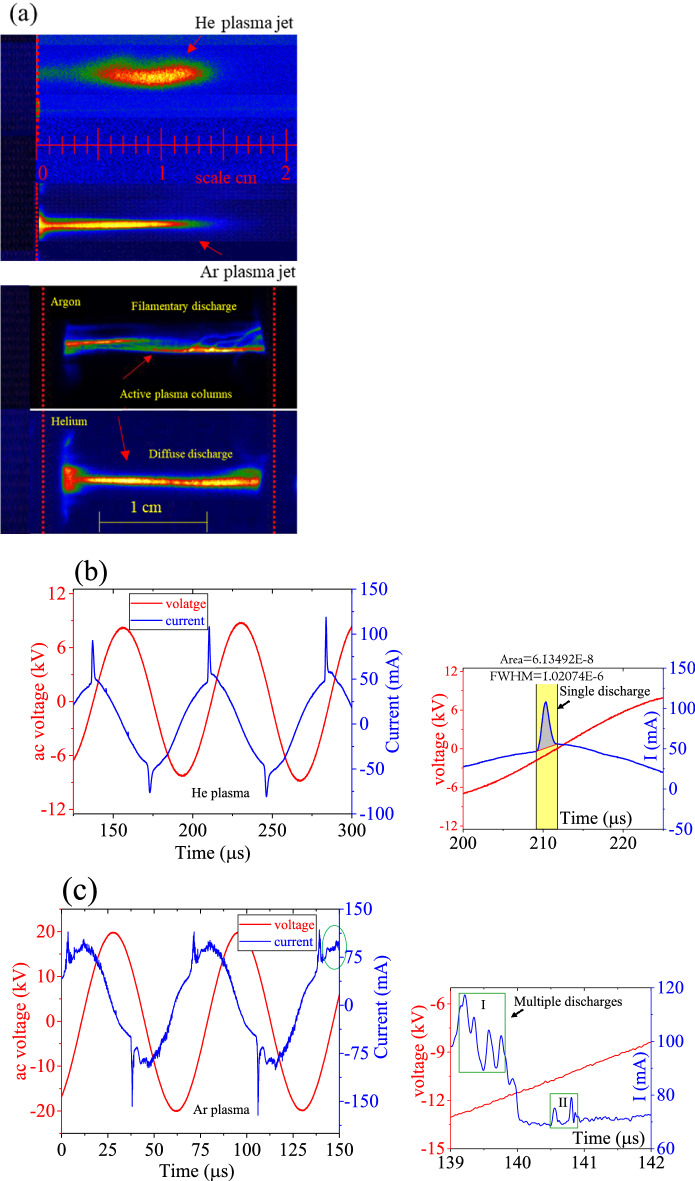
iCCD optical images discharge jets & active plasma columns (**a**), and waveforms of the current–voltage signals (**b**) of argon and helium plasmas generated at 10 lit. min^-1^ in the tubular geometry in the normal mode operation. The enclosed multiple current peaks in the green circle signposts filamentary behaviour of argon discharge. The insets in (**b**) and (**c**) are enlarged portions, representing the single and multiple discharges of helium and argon respectively. The area under the curve in the inset of (**b**) represents the total amount of charge in the single discharge per half cycle of the ac voltage.

Figure 2a shows optical images of the external afterglow (eAG) discharges, protruding from the tube orifice as visible plasma jets (top image) and the active zone (AZ) inside the tube enclosed by the electrodes and is expressed as the active plasma columns (bottom image). The active plasma volume, bounded by the discharge region between the electrodes is about $361.911{ } \times 10^{ - 3} {\text{ cm}}^{3}$. Consistent with the previous reports^5,28,39–42^, the discharge is diffusive for helium and filamentous for argon discharge as can be seen in Fig. 2a. The eAG discharge is the transferred plasma jet, formed by the charge overflow and propagates in a channelized region in a straight line in the gas flow. For both argon and helium, the jet length is ⁓ 15 mm. The physical appearance of the helium jet has a close resemblance to a knife or sword whereas slightly a needle-type jet with a sharp tip is formed in argon gas. Downstream the propagation direction, the rapidly decreasing ac fields, and the higher electrical breakdown strength of the air obscure the jet to get narrow as it penetrates the ambient gas. Figure 2b,c show the instantaneous signals of the HV probe and the discharge current in the normal electrode arrangements. For helium, a single current peak appears in each half-cycle of the ac voltage and sequentially repeats at intervals of 36 µs. Such plasma behaviour represents a diffused character of the discharge^43^. On the other hand, the multi-peak character of the current pulses in argon represents a streamer-like or filamentary discharge^28,39–42^. The filamentous character is more obvious relatively at slower flow rates and the plasma jet shrinks while at faster gas flow rates, the filaments are less or disappear and discharge seems homogeneous^39–42^. For argon, the first current pulse represents primary discharge filaments while the secondary peaks correspond to secondary discharges. On average, the multiple discharges are spatially separated by 230 ns. Since the discharge time is much smaller than the exposure time of ms, so the images are superimposed by many discharge filaments in each current pulse and make their physical appearance incessant. The regions of the side discharges are identified by the enlarged current signals in the inset of (b). The number of electrons per half cycle estimated for the positive half cycle of the ac voltage is large by a factor of 10 for helium $\left( {{\text{n}}_{{{\text{He}}}} = {\text{q}}/{\text{e}}} \right)$ 3.72 × 10^11^ > ${\text{n}}_{{{\text{Ar}}}}$ = 7.2 × 10^10^. Argon discharge filaments are highly conducting (J ~ 10^2^–10^3^ A cm^−2^) and characterised by a highly concentrated electric field (E = 10^7^ V/m), and high electron density (10^14^–10^15^ cm^−3^) compared to electron charge density of 10^10^–10^12^ cm^−3^ in the glow or diffuse discharges^2,10,12–14,20^. The filamentary type discharge is rather transient wherein the short current pulses are randomly appeared and sustained for the period of 10–100 ns with the typical heat energy of 100 µJ per microdischarge^10,12–15,20^. The discharge filaments usually remain in contact with the dielectric surface and dissipate heat energy and for this reason, it is avoided for treating thermally sensitive biological tissues and polymeric materials.

**Figure 3:**
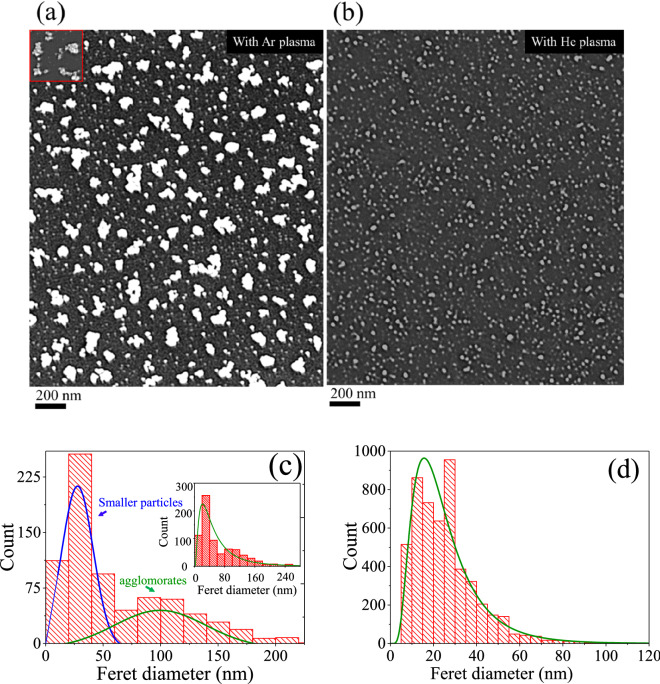
SEM images of nanoparticle aerosols processed with (**a**) argon and (**b**) helium plasma jets and collected downstream the tube on a silicon substrate. Feret diameter distributions are given in (**c**, **d**). The inset in (**a**) is a small patch at a different contrast while the inset in (**c**) is the overall distribution for the whole surface.

**Figure 4:**
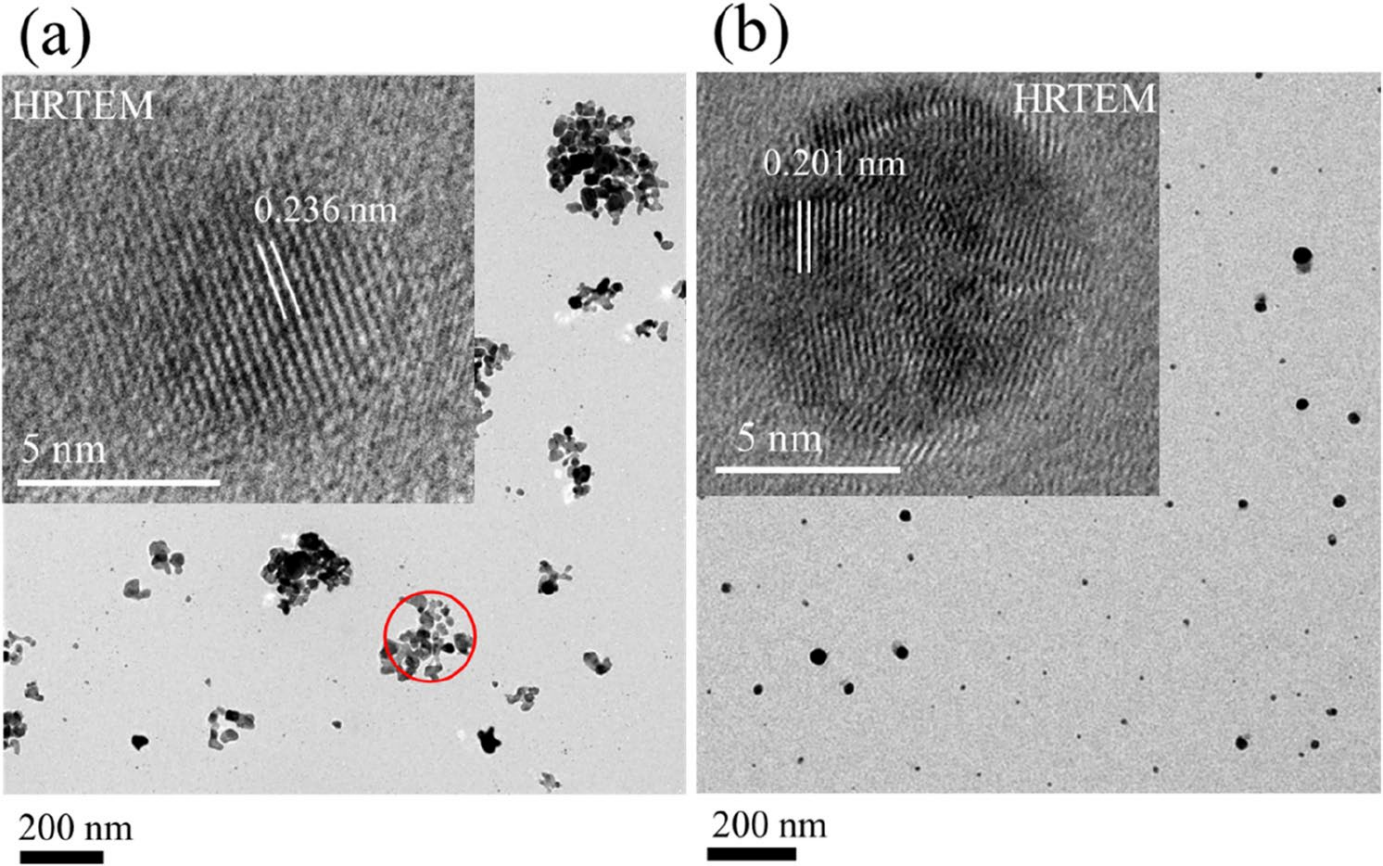
Bright-field TEM images ofnanoparticle aerosols on amorphous-carbon film coated Cu- TEM- grids, obtained with argon (**a**) and helium (**b**) plasma jets at atmospheric gas pressure. Insets are the HR-TEM images of a single crystalline particle showing the lattice fringes and the red circle in (**a**) represents a cluster of assembled particles.

**Figure 5:**
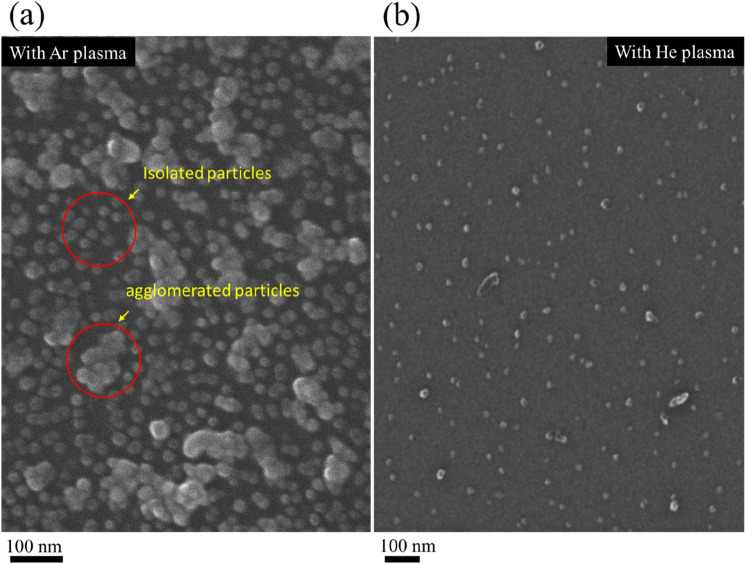
Helium ion microscopy representative images of silver nanoparticle aerosols, collected on Si with discharge jets, excited in argon (**a**), helium (**b**). The red circles drawn in (**a**) is to indicate isolated particles and agglomeration formed by the argon plasma jet. The images were taken with a GFIS field of view of 1 µm and an accelerating voltage of 30 kV with a scan dwell time of 1 µs.

**Figure 6:**
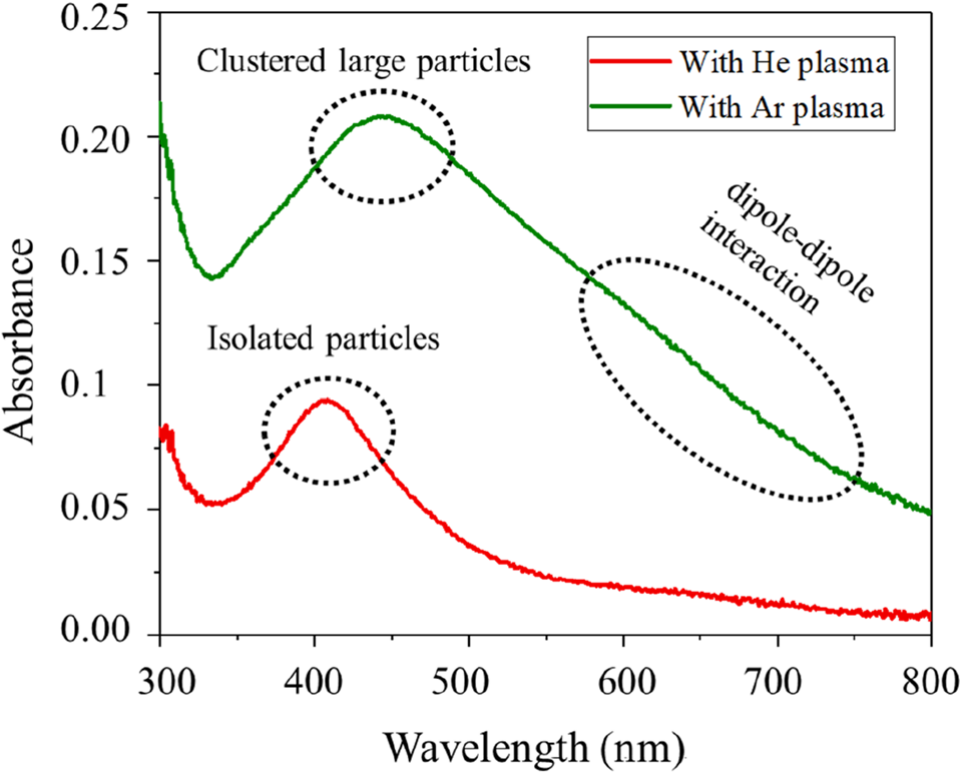
Surface plasmon resonance of silver nanoparticle aerosols entrained with the discharge jets of argon and helium and deposited on a glass slide. The doted circles indicate various plasmonic features of the deposited aerosols.

**Figure 7:**
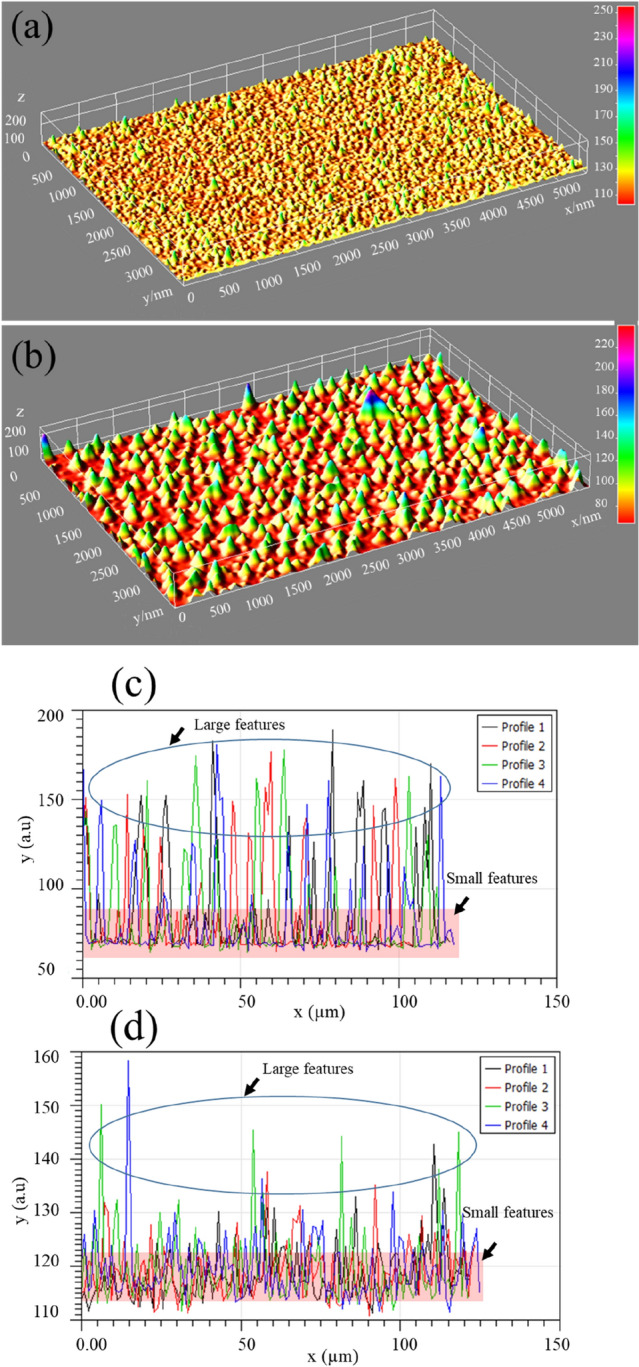
Interactive 3D-surface plots constructed for images shown in (**a**) and (**b**) of Fig. 3 and the surface line profiles, drawn over different regions (**c**, **d**). The luminance of the image is interpreted as height for the plot and is indicated by the colour scale bars to make an estimation of the particle dimension. The highlighted regions and the circles indicate the smaller and larger features of the particulate surfaces formed by the discharge jets.

Figure 3 illustrates SEM images (a, b) and particle size distributions (c, d) of the nanoparticle films on Si, obtained with argon and helium plasma jets in the normal mode operation. In both cases, there is a significant difference in the morphological features and spatial distribution of the particles. An argon plasma jet preferentially formed aggregated structures together with the smaller particles, dispersed in the background of the large clusters. These particles seem to adhere together through the surface physical interaction while retaining their physical identity as indicated by the small patch in the inset of Fig. 3a. Compared to the previous results reported in ref. 27, 35, for the increased gas flow rate, apart from the large clusters (formed by the smaller size particles of dimension in the range of 5–30 nm), there is a sufficient population of smaller size particles in the background of the large clusters and so presents a bimodal distribution (Fig. 3c). The angle average Ferret diameter of the large aggregates is ⁓ 90 (24) and that of the smaller particles ⁓ 28 (15) nm as shown in Fig. 3c, with values in the bracket denote standard deviation of the particle distribution. The average Feret size estimated for the overall distribution on the whole surface was ⁓ 63 (53) nm as given in the inset of (Fig. 3c). Contrary to this, the helium plasma jet leads to spatially well-dispersed particles with the mean angle average Feret diameter of ⁓ 26 (12) nm. Interestingly, the sparsely distributed faceted features previously reported in ref. 27, were not observed here. This envisages that for the specific discharge condition and gas flow rate, the surface nanofeatures can be tailored. For both plasma jets, the particles are 3–4 times smaller than the large clusters formed by an argon plasma jet. The distribution is narrow, spanning over the region of 5–50 nm, and indicates that in the ionised gas jets, the smaller particles are almost of the same size. With Ar- plasma jet, the non-uniform surface previously described in Fig. 3a is formed due to unequal distribution of the large-size clusters and is strongly supported by the surface line scans displayed in Fig. 7a–c. The deposited nanomaterial in both cases was particulate and plasmonic active, showing a prominent absorbance due to surface plasmon resonance (SPR) as described in Fig. 6. For the large clusters, the widely spanned absorbance over the visible region with a long stretched tail in the infra-red region indicates the interactive behaviour of the physically coupled particles. Also, SPR maxima shifted from ⁓ 390 (for helium) to ⁓ 410 nm (for argon) and exhibited a broad-band feature that strongly depends on the particle size, shape, and distribution^32,36^. The amplitude of SPR is markedly higher for the deposit of the argon plasma jet: this indicates that an argon plasma jet entrains the particle aerosol more efficiently compared to the helium plasma jet. This aspect could be linked with a well-known phenomenon of electro-hydro-dynamic gas pumping the so-called ionic wind or electric wind, induced in the high-fields regions of the discharge^44,45^. The crystalline nature, morphology, and formation of agglomerates were further investigated by TEM, HR-TEM, and HIM, and images are displayed in Figs. 4 and 5 respectively. These results clearly support the formation of large clusters with argon plasma jets (as indicated by the red circles). The fine fringes in the HR-TEM images in the insets of Fig. 4a,b, and the lattice spacing 0.236 nm, and 0.201 nm, assigned to (111) and (200) planes of silver respectively specified a crystalline nature of the particle (Fig. 6).

**Figure 8:**
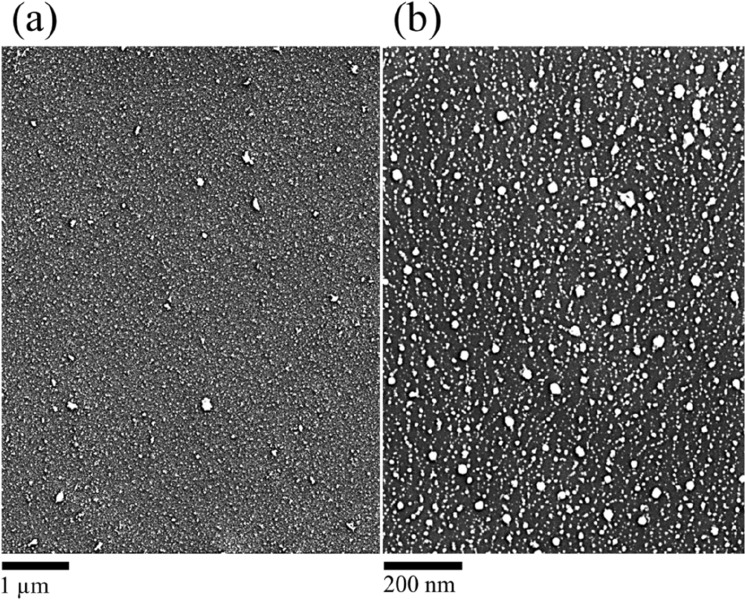
SEM images showing surface nanofeatures of Si, covered with silver nanoparticles using a helium plasma jet, launched in the reversed mode by setting the live electrode downstream and ground upstream of the reactor while the rest of the plasma conditions persist.

Interesting 3-dimensional interactive 3D-surface plots and the surface line scans, drawn over different regions of the surface are displayed in Fig. 7a–d respectively. The bumpy and nutted surface for argon plasma and the smart nanofeatures in the case of helium plasma jet strongly support our previous observation. The luminance of the image is interpreted as height for the plot as indicated by the colour scale bars to make an estimation of the particle dimension. An interesting 50 mm long plasma jet was launched with the helium plasma jet by reversing the positions of the electrodes such that the upstream electrode is grounded and the electrode downstream the tube is live (not shown here). In this case, there was a significant increase in the surface coverage as presented in Fig. 8a,b. Though there is a significant increase in the amount of deposit (particles) on the surface, no such pronounced agglomeration occurs which signifies that a helium plasma stream does not facilitate the formation of particle aggregates. It is expected, compare to the normal mode operation, in the reversed electrodes arrangement, a sufficiently large electric-wind effect is induced and hence leads to such an enhanced deposition. This elucidates that the surface features and subsequently the particle coverage can be tuned to obtain a sparsely and or densely populated particulate surface by changing the plasma operational conditions. It is noteworthy that the aggregate behaviour of silver nanoparticles is of high significance in connection to sensing, antibiotics in marine biology and many more. To stay within the framework of the study, here, we briefly discuss the SERS aspect of these nanostructures in the context of particle aggregates.

**Figure 9:**
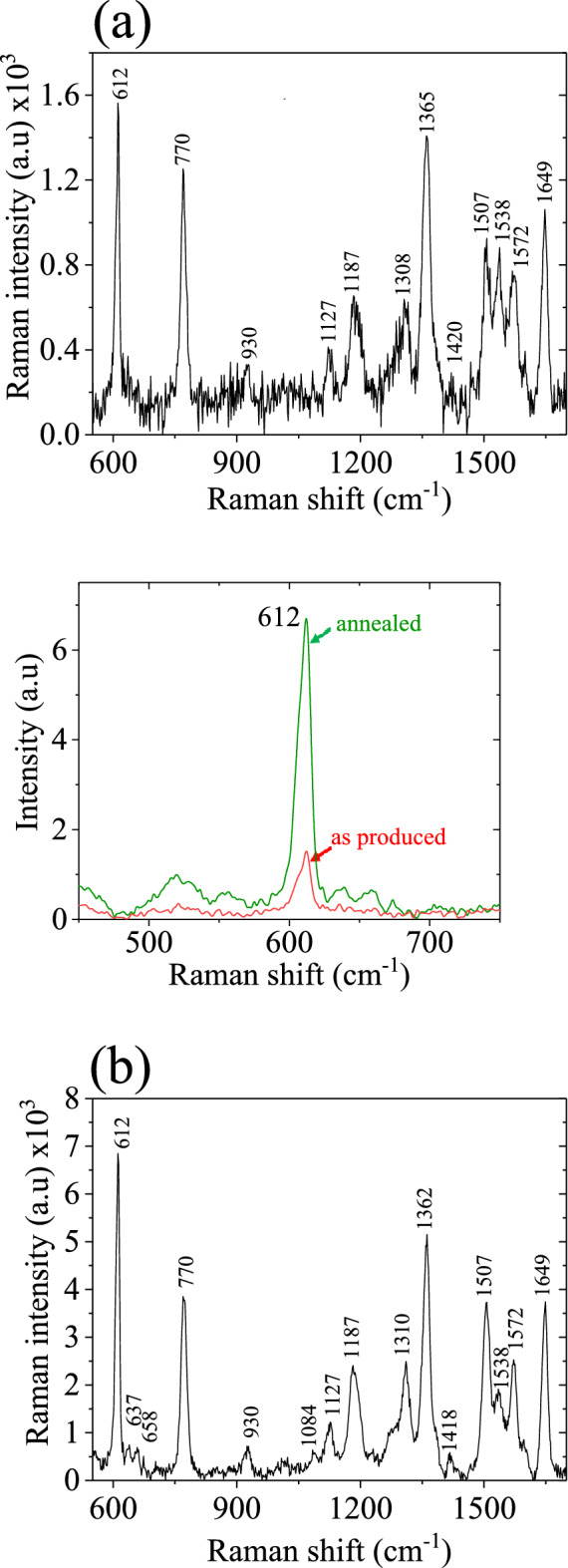
SERS spectra of Rh6G (10^−5^ M), taken by using a laser excitation wavelength of 532 nm, power ⁓ 10 µW, and integration time of 3 s on glass surfaces covered with plasmonic metal aerosols of silver, deposited by the plasma jets of; (**a**) helium, and (**b**) argon. The inset shows a zoom portion of the SERS spectra of the characteristic peak of 612 cm^−1^ for quantification of the SERS response.

The SERS sensitivity aspect was explored for a 10^−5^ M stock aqueous solution of an organic fluorescent dye Rhodamine 6G (Rh6G) for comparison purposes to understand the SERS effect in both cases. Measurements were taken in a broad range from 600 to 1600 cm^−1^, a region that spans most of the transparency window of these chemicals. Briefly, the sample was prepared by drop casting 10 micro-litters of Rh6G solution directly onto the surface covered with the particle aerosols. The Raman measurements were performed using a Horiba Jobin Yvon LabRAM HR800 spectrometer equipped with a CCD detector of spectral resolution 4 cm ^-1^. The excitation wavelength (532 nm) was focused on the sample surface down to ~ 1 µm with a × 100 objective lens and the power of the laser was set to ~ 200 μW using a density filter. The spectrum position was calibrated to the 520.221 cm^−1^ peak of a Si/SiO_2_ substrate before the acquisition and the photoluminescence background in the Raman spectra was fitted with a polynomial of degree 8 and subtracted from the data. The SERS spectra measured are shown in Fig. 9a,b. The 612 cm^−1^ peak of Rh6G, separately shown in the zoom spectra of the inset of Fig. 9b is taken to quantitatively estimate and compare SERS detection sensitivity in both cases. The noise value was approximated to choose at 590 cm^−1^ to calculate signal-to- noise ratio (SNR) and apparent enhancing factor (AEF) defined by ${\text{SNR}} = \frac{{{\text{S}} - {\text{N}}}}{{\sqrt {\text{N}} }}{\text{ a}}$ nd ${\text{AEF}} = \frac{{{\text{I}}_{{{\text{SERS}}}} }}{{{\text{I}}_{{{\text{ref}}}} }} \times \frac{{{\text{C}}_{{{\text{ref}}}} }}{{{\text{C}}_{{{\text{SERS}}}} }}{ }.{ }$ Where S is the signal intensity and N is the noise intensity,${\text{ I}}_{{{\text{SERS}} = }}$ integrated SRES intensity of 612 cm^−1^ Raman band,${\text{ I}}_{{{\text{ref}} = }}$ the integrated intensity of the same Raman band of reference molar solution Rh6G (10^−2^ M), ${\text{C}}_{{{\text{SERS}}}} =$ is the number of probe molecule R6G (10^−5^ M), adsorbed on SERS substrate, ${\text{C}}_{{{\text{ref}}}} =$ is the number of probe molecule of the reference molecular solution R6G (10^–2^ M). The SNR of $1.07 \times 10^{2}$ and AEF of ${ }1.82 \times 10^{3}$ of the particles obtained with the helium plasma compared to SNR $2.62 \times 10^{2}$ and AEF $7.98 \times 10^{3} { }$ of the agglomerated nanostructures, formed by an argon discharge jet marked a relatively large sensitivity and elegant SERS performance. In the latter case, plasmonic nano-junctions formed by the particle aggregates act as smart ‘hot sites’ for the probed molecule where the electric field is magnified, and as a result, the SERS effect is enhanced. This happens because a larger size particle trumps the loss in surface coverage and greater SERS signals are achieved. In the case of argon discharge, agglomerates undermine spot-spot signals reproducibility and could be a common barrier for SERS substrates based on the particle aggregates to achieve reproducible detection quantitatively. But this is not surprising and is expected for the unequal spatial distribution of the nanostructures on the surface. This result has marked that instead of the fact that agglomerates deteriorate SERS reproducibility, still are useable as effective candidates for practical purposes in SERS. On the other hand, SERS signals for the helium plasma-made substrate are relatively weak but fairly reproducible due to the relatively superior spatial distribution of the particles. The different enhancement observed for the different peaks of the optically active phonon modes is due to the short-range coupling between the inter-connected particles^26,31^. Also, the electromagnetic field in the particle junctions is not a simple coherent sum of the fields from the individual particles and this may lead to different enhancements. Instead, as the particles approach each other to make a surface connection, there is a dramatic enhancement factor increase of more than 5 times for certain bands (i.e., 612 cm^-1^). Likely, previous reported studies, aggregates of nanosilver are good plasmonic active substrates and most of the spectral regions was justly appraised and proved by the results^31,46^.

## Interpretation of results

The generation and processing of a nanomaterial in cold plasma is a complex scenario of mesoscopic physics. The presence of an ionised gas medium (the gas discharge) inevitably produces a continuous flux of ions/electrons and enhances the growth phase of a nanomaterial for surface deposition. To understand the underlying physics of the observed findings, it is essential to consider the gas discharge together with the particle aerosols as the foreign material as a whole a dusty plasma system.

For DBD cold plasma jets, we know that the degree of ionisation is much smaller $({\upchi } < 10^{ - 3} - 10^{ - 8} ){\text{ or} \nu }_{{\text{ei }}} < {\upnu }_{{\text{en }}} ,$ where ${\upnu }_{{\text{ei }}}$ and ${\upnu }_{{\text{en }}}$ are the electron–ion and electron-neutral collision frequencies. In such discharges, the growth of a nanomaterial is the preliminary phase of nucleation, followed by particle-plasma interaction, processing, and subsequent surface deposition. On average, the classical model of ions collection by the floating particle aerosol in the discharge holds good, however, the attraction of particles is dominated by the instantaneous value of the total charge which is subject to a large fluctuation. In such a condition, the electrostatic agglomeration has always been insufficient in expounding the phenomenon, and in most cases, the charge fluctuation is coupled to the plasma turbulence and oscillations^47^.

**Figure 10:**
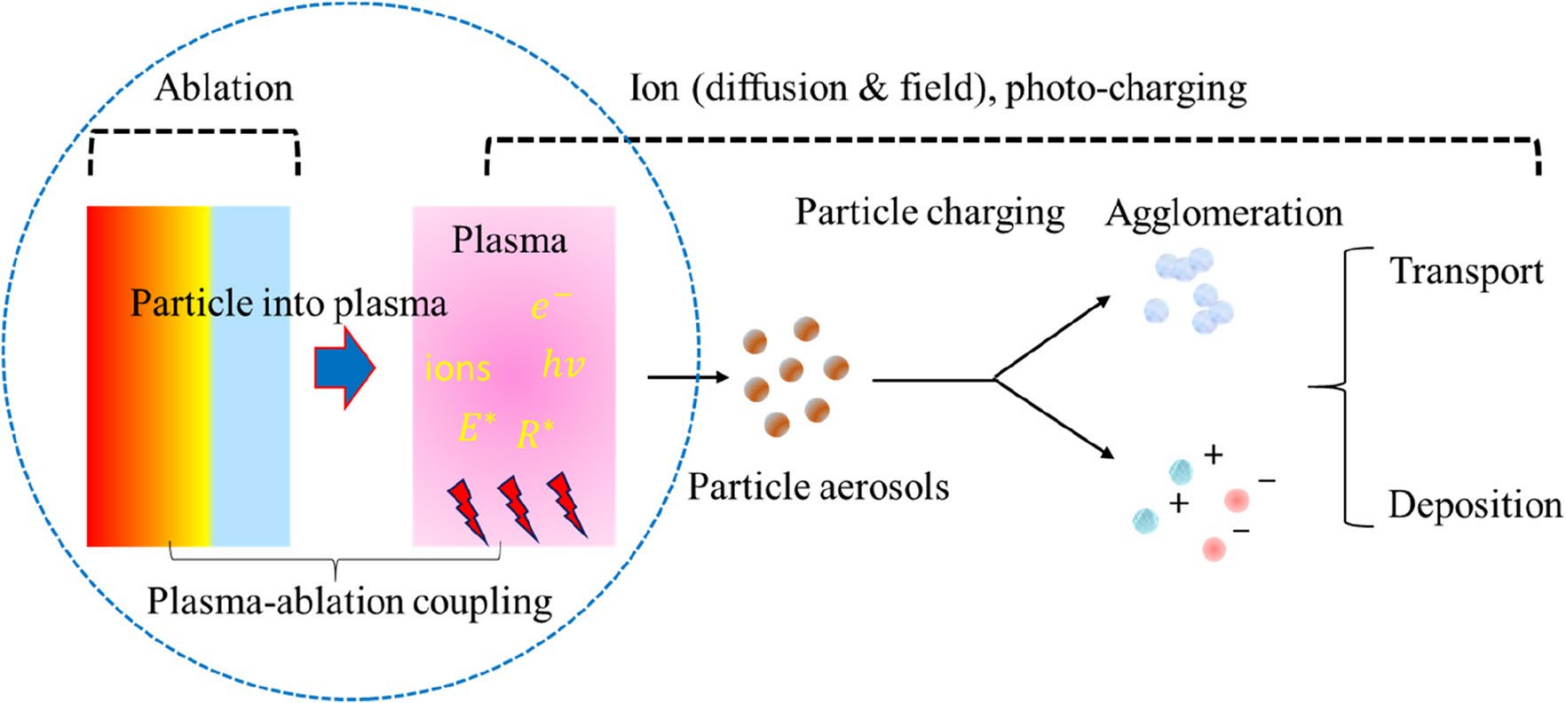
Schematic showing the mechanisms of plasma-ablation plume coupling, charging of particle aerosol in the plasma, and agglomerates formation.

The enhanced particle surface energy, particle charging, and ionic wind are the induced progressions of DBDs and strongly depend on the nature of the discharge gas. In the concept of dusty plasma, the charge level of the particle aerosol depends on the electric field and the product of ion density and resident time in the plasma stream i.e. $${\text{n}}_{{{\text{ion}}}} \left( {{\text{cm}}^{ - 3} } \right) \times {\uptau }\left( {\text{s}} \right)$$ as described in ref.^47^. On the other hand, the phenomenon of plasma ionic –wind promotes momentum in the bulk flow of the feed gas^44,45^ and leads to a faster surface deposition. We noticed, in argon and helium, discharge follows different mechanisms and is filamentary in argon and diffusive type in helium. For both discharges, the electron population as well as overall energy/power dissipation are thus unlike. The power (P) dissipation in the discharge is described by $${\text{P}}_{{{\text{e}},{\text{i}}}} = {\upsigma }_{{{\text{e}},{\text{i}}}} {\text{E}}^{2}$$ (where $${\upsigma }_{{{\text{e}},{\text{i}}}} = {\text{en}}_{{{\text{e}},{\text{i}}}} {\upmu }_{{{\text{e}},{\text{i}}}}$$ is defined as the electrical conductivity)^6^. Since in the plasma, charge concentration remains the same i.e. $${\text{n}}_{{\text{e}}} = {\text{n}}_{{\text{i}}}$$, mobility $$({\upmu }_{{{\text{e}},{\text{i}}}} )$$ of the charge species is defined by $${\upmu }_{{{\text{e}},{\text{i}}}} = \frac{{\text{e}}}{{{\text{m}}_{{{\text{e}},{\text{i}}}} {\upnu }_{{{\text{e}},{\text{i}}}} }}$$ (where $${\upnu }_{{{\text{e}},{\text{i}}}}$$ is the collision frequency) and $${\upnu }_{{\text{e}}}$$ is typically by 2–3 orders of magnitude larger than the $${\upnu }_{{\text{i}}} { }$$ but at the same time $${\text{m}}_{{\text{i}}}$$ exceeds that of $${\text{m}}_{{\text{e}}}$$ by 4–5 orders of magnitude. This makes the expression that $${\text{P}}_{{\text{e}}} \approx {\text{P}}_{{\text{i}}} \left( {10^{2} - 10^{3} } \right)$$ and consequently $${\raise0.7ex\hbox{${{\text{T}}_{{\text{e}}} }$} \!\mathord{\left/ {\vphantom {{{\text{T}}_{{\text{e}}} } {{\text{T}}_{{\text{i}}} }}}\right.\kern-0pt} \!\lower0.7ex\hbox{${{\text{T}}_{{\text{i}}} }$}} = 10^{2} .$$ This clearly marks that the major part of the driving ac power in the discharge is absorbed by the plasma electrons while a fractional part is taken by the ions; keeping the electrons to stay at a much higher temperature than the ions. The higher electron temperature comes from the strong coupling of the impressed ac field to the electron due to their less mass and higher mobility^6^. Since, helium discharge occurs at a much lower breakdown voltage than argon discharge and the discharge presents a moderate number of electrons (Fig. 2b,c). The higher-excitation voltage of argon yields multiple discharges and defines its filamentary behaviour (Fig. 2a. One aspect of such discharge filaments is to dissipate a significant amount of energy in the discharge gas^2,6^. The localised heating makes the plasma environment favourable to enhancing the particle surface energy and facilitating the mechanisms of agglomerates formation^48^. Another aspect is the interaction of plasma with the particle aerosol and subsequently the charging mechanisms (see Fig. 10) and is rather a complicated process in view of its multiphase hydrodynamics with ionised gas as the continuous phase and the solid metal particle as the dispersed phase^49–51^. Briefly, dust “nanoparticle” accumulates charge by capturing both electrons and ions from the ambient plasma, however, initially capture electrons at a faster rate than ions, due to their lighter mass and faster moment. The particle therefore overall acquires a net negative charge and generates a net electrostatic repulsion among the particles: this force leads the particles to spatially disperse likely we observed in the case of helium plasma jet. The orbital motion limited (OML) model^51^ (originally derived for the Langmuir probe measurements) and the numerical modelling of multipole distribution by Matthews et al*.*^52^ have further explained the phenomenon. By OML theory, a nanoparticle immersed in the plasma is surrounded by an electrostatic potential and around it, the electrons, and ions in the plasma travel on collisionless trajectories. In the case of positive ions, angular momentum is taken into account as it adds a repulsive component to the attractive electrostatic potential and particles are less negatively charged. The underlying charging mechanisms are strongly affected by the particle properties, the plasma electric field, and the discharge conditions. Preliminary studies suggest that the diffusion charging mechanism dominates particle aerosol with a size below 100 nm, and the field charging governs aerosol larger than 1 μm^49^. UV optical emissions from the plasma also affect the particle charging dynamics. In the helium discharge jet, the agglomeration process is suppressed by the electrostatic repulsion force for the unipolar charged particle aerosol^53^. For the bipolar charged aerosol, the agglomeration process is enhanced because of electrical forces such as ion‐induced Van der Waal's force and image force, and Columb attraction which was observed for argon discharge jet^49,54^. Since electrons are relatively more mobile and favourably follow the high ac field relatively faster. This enhances the possibility of the electrons attaching to the particle surface (on average, 10–50 electrons are possible on an isolated grain subject to the size of the particle) to generate a repulsive Coulomb force to hinder agglomerates formation^53,54^. But these are short-range forces, and don’t pronouncedly operate and even become less effective at particle separation. In such a scenario, the particle surface energy prevails effectively and favourably permits the phenomena of agglomeration by the surface bonding likely observed with the argon discharge jet. In the helium discharge jet, it is expected that the large population of electrons leads to the possibility of generating an electrostatic coulomb repulsion, sufficiently larger than the conventional van der Walls forces, and as a consequence particles are spatially dispersed. It is worth mentioning that in an ionized medium, particle charging strongly depends on the particle size as well as prior existing coupled particles^54,55^. A smaller size particle (size < 100 nm) can diffuse more effectively and hence charging by the drift of ions remains dominant^56^. A combined field-diffusion charging mechanism becomes effective for the large size particles (sub µm & µm)^57^ and doesn’t fit our present case.

Considering the effects of plasma ionic wind, it generates in the regions of the high electric field of the discharge and causes an induced bulk flow in the gas medium by imparting momentum to the gas particles which is described by ${\text{u}} = \left( {\frac{{{\upvarepsilon }_{{\text{o}}} {\text{E}}^{2} }}{{\uprho }} + {\text{u}}_{0}^{2} } \right)^{1/2}$^58^ where, ${\text{u}}$ is the induced bulk velocity with the discharge on, $\varepsilon_{o}$: permittivity of free space, ${\text{E}}$: electric field in the plasma, ${\text{u}}_{{\text{o}}}$: gas flow velocity when discharge is off, and ${\uprho }$: gas density. Since ${\text{ u}}_{{\text{o}}} ,{{ \varepsilon }}_{{\text{o}}}$ are constant and $\propto \frac{1}{{\uprho }}$ $,{\text{u}} \propto {\text{E}}^{2}$, this means though, ${\uprho }_{{{\text{Ar}}}} > {\uprho }_{{{\text{He}}}}$, due to the significantly higher electric field in argon discharge, the induced ionic-wind effect is relatively large. This suggests that an argon plasma jet has a comparatively large efficacy of entraining the particle aerosol at the ambient condition than a helium plasma jet. This effect perturbs the charge equilibrium to the particle by the discharge current and the negative surface charge effect decreases simultaneously. As a result, electrostatic repulsion is undermined and agglomerates formation is promoted. The reverse effect is true for the helium discharge wherein the formation of particle aggregates is hindered and particles are spatially dispersed. This fits in the framework of a relatively smaller ionic-wind effect of helium discharge which generates a low collisional environment. Under this unfavourable condition, particle surface energy doesn’t enhance and the role of Coulomb’s repulsion force becomes dominant. The plasma-induced ionic-wind effect is measurable in terms of flow velocity by a dielectric pitot tube to supplement the experimental findings quantitatively. In near future, this aspect of the gas discharge could be one of our interesting studies to demonstrate it experimentally in connection with the plasma assists particle post-processing and its subsequent deposition.

## Conclusion and outlook

Our research highlighted nonthermal plasma post-processing and deposition of plasmonic nanoparticle aerosol of silver for surface functionality by working with the filamentary and diffused discharge jets of argon and helium from a DBD plasma source. The interaction of the discharge jets with the particle aerosol led to unlike surface coverage, morphological features, and detection sensitivity. With the argon discharge jet, particles were clustered preferentially in large agglomerates whereas spatially well-dispersed particles were obtained with the helium discharge. Argon plasma jet showed relatively a large efficacy of entraining the particle aerosol due to its upshot of induced high-electric wind. The nanostructures in each case were crystalline and plasmonic active, showing enhanced SERS sensitivity as a test of Rh6G (10^−5^ M). The SERS enhancement factor for the assembled particulates, formed by the argon discharge jet was comparatively large. For helium discharge, the coulomb repulsion is predominant while for argon plasma, the high energy dissipation and plasma-induced momentum predominantly promoted agglomerates formation. Further study on the charging of particle aerosol while floating in nonthermal DBD plasma jets and its dependence on the particle size is necessary to discern the underlying mesoscopic physics with more clarity. A better outlook seems modeling and simulation to come up with this research problem. It is noticed, processing a nanomaterial with aid of nonthermal plasma jets has a strong potential to feed into surface nanoengineering, coating technology, sensor, catalysis, and the pharmaceutical industry.

## Data Availability

The design and operation of the DBD plasma setup used in conjunction along with the APLD and any data described in this paper and support the findings of this study is available from the corresponding author upon reasonable request.
