# Author Correction: Processing laser ablated plasmonic nanoparticle aerosols with nonthermal dielectric barrier discharge jets of argon and helium and plasma induced effects

**DOI:** 10.1038/s41598-023-29696-5

**Published:** 2023-02-13

**Authors:** Taj Muhammad Khan, Gustavo Andrade Silva Alves, Amjad Iqbal

**Affiliations:** 1 School of Physics and CRANN, Trinity College Dublin, The University of Dublin, Dublin 2, Ireland; 2 National Institute of Lasers and Optronics College, Pakistan Institute of Engineering and Applied Sciences, Nilore, Islamabad 45650 Pakistan; 3 Department of Materials Technologies, Faculty of Materials Engineering, Silesian University of Technology, 44-100 Gliwice, Poland; 4 Department of Mechanical Engineering, CEMMPRE- Centre for Mechanical Engineering, Materials and Processes, University of Coimbra, Rua Luis Reis Santos, 3030-788 Coimbra, Portugal

Correction to: *Scientific Reports* 10.1038/s41598-022-27294-5, published online 03 January 2023

The original version of this Article contained errors in the Acknowledgements section.

"This research was financially supported by Science Foundation Ireland (SFI) under Investigator Project 12/IP/1662 and the excellence initiative –Research university program implemented at the Silesian university of technology, one year 2022 under the project No. 32/014/SDU/10-22-25. We greatly acknowledge the lab support of Dr. James Creel in iCCD imaging."

now reads:

"This research was initiated and directed by Dr James G Lunney. This research was supported by a grant from Science Foundation Ireland (SFI) under Investigator Project 12/IP/1662, where Dr James G Lunney was the principal investigator, and the excellence initiative –Research university program implemented at the Silesian university of technology, one year 2022 under the project No. 32/014/SDU/10-22-25. We greatly acknowledge the lab support of Dr. James Creel in iCCD imaging, as well as Dr Katarzyna Siewierska’s help with Raman spectroscopy."

The original Article has been corrected.

